# Testing necessary regional frontal contributions to value assessment and fixation-based updating

**DOI:** 10.1038/ncomms10120

**Published:** 2015-12-14

**Authors:** Avinash R. Vaidya, Lesley K. Fellows

**Affiliations:** 1 Department of Neurology and Neurosurgery, Montreal Neurological Institute, McGill University, 3801 University Street, Montreal, Quebec, Canada H3A 2B4

## Abstract

Value-based decisions are biased by the time people spend viewing each option: Options fixated longer are chosen more often, even when previously rated as less appealing. This bias is thought to reflect ‘value updating' as new evidence is accumulated. Prior work has shown that ventromedial prefrontal cortex (PFC) carries a fixation-dependent value comparison signal, while other studies implicate dorsomedial PFC in representing the value of alternative options. Here, we test whether these regions are necessary for fixation-related value updating in 33 people with frontal lobe damage and 27 healthy controls performing a simple choice task. We show that damage to dorsomedial PFC leads to an exaggerated influence of fixations on choice, while damage to ventromedial or lateral PFC has no effect on this bias. These findings suggest a critical role for dorsomedial, and not ventromedial PFC, in mediating the relative influence of current fixations and a priori value on choice.

## Abstract (web summary)

In value-based decisions, the longer one fixates on an option, the more likely one is to choose it. Here, the authors compare the performance of people with focal frontal lobe damage in a simple choice task and show that damage to the dorsomedial PFC leads to exaggerated fixation-related value updating.

## Graphical abstract

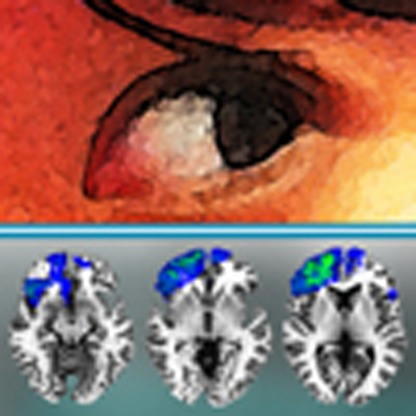

Traditional theories of economic decision-making argue that a rational actor makes choices guided by a comparison of the utility (or subjective value) of available options, leading to internally consistent choices^1^,^2^. However, humans make decisions more flexibly, expressing a variety of biases. Recent studies have shown that visual fixations influence value-based choices: subjects choose options they have looked at longer more often than would be predicted by their a priori value ratings of those options alone^3^,^4^,^5^,^6^. This bias is present even when the duration of fixations is experimentally manipulated^7^,^8^. These findings argue that decisions do not rely only on a comparison of the pre-determined values of options, but are also influenced by information gathered ‘in the moment' through fixations.

We know very little about the neural processes underlying this dynamic value updating. However, regions within the frontal lobes have been implicated in value-based choice more generally. Activity within ventromedial PFC reflects the subjective value of available options^9^,^10^,^11^,^12^, and predicts choice^13^. Patients with damage to this region and adjacent orbitofrontal cortex (together termed ventromedial frontal lobe; VMF) are more internally inconsistent when making preference-based choices^14^,^15^,^16^. Macaques with medial orbitofrontal cortex lesions fail to update the value of visual cues in selective satiety paradigms^17^. These findings have together been taken as evidence that VMF represents and compares options in a common value currency^10^,^12^,^18^.

Dorsomedial frontal (DMF) regions have also been linked to value processing and decision-making. Dynamic value-related signals have been reported within this region in functional magnetic resonance imaging (fMRI) and electrophysiology studies, and linked to choice, particularly in foraging contexts^11^,^19^,^20^. Lesions to this region in humans and macaques lead to impairment in optimal action-value learning^21^,^22^, but whether this region is critical for decision-making under more ecologically valid conditions remains unclear.

Neural representations of value are dynamically modulated as a decision is prepared. Correlates of accumulating value information have been found in PFC before choice, suggesting that values are constructed in this region during the decision process^23^,^24^,^25^,^26^,^27^. Lim *et al*.^28^ showed that hemodynamic signals reflecting relative value in PFC were dependent on which option the subject looked at when choosing between foods, arguing that these value computations were influenced by fixations. However, whether these signals are necessary for value updating during decision-making has not been established.

Here, we test the causal role of three PFC subregions in this fixation-driven dynamic value updating. Patients with damage to ventromedial (VMF), dorsomedial (DMF) or lateral frontal (LF) lobes, and age-matched healthy control subjects judged how much they wanted a variety of artworks, and then chose between pairs of these artworks while we tracked their eye movements. As in prior studies with this paradigm, subjects' choices are biased by fixations. DMF damage leads to an exaggerated influence of fixations on choice, whereas VMF- and LF-damaged subjects perform normally in this and most other aspects of the task.

## Results

**Figure 1:**
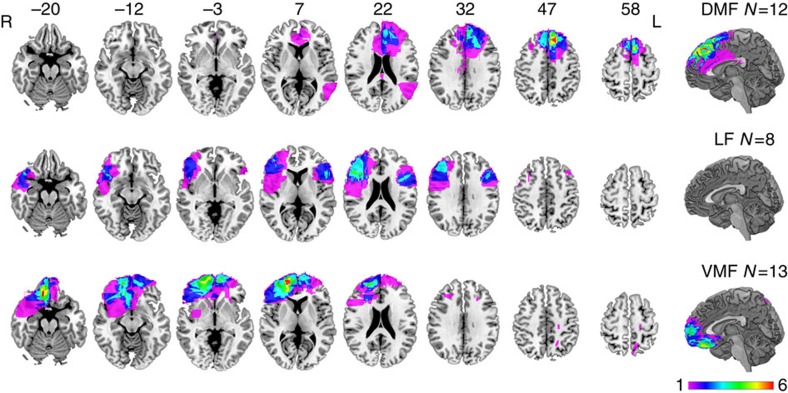
Representative axial slices and mid-sagittal view of the MNI brain showing extent of lesion overlap in frontal damaged groups. Rows show lesion overlap in the dorsomedial frontal (DMF), lateral frontal (LF) and ventromedial frontal (VMF) groups. Numbers above slices indicate *z* coordinates of axial slices in MNI space. Colours indicate the extent of lesion overlap, as indicated by the colour scale. L, left; R, right.

People with focal lesions involving the frontal lobes (*N*=33) and healthy older controls (*N*=27) were recruited from the Cognitive Neuroscience Research Registry at McGill University. PFC patients were divided a priori into three subgroups, (VMF, DMF and LF), based on the location of their damage, assessed on their most recent MRI or CT imaging by a neurologist blind to task performance, according to standard boundaries^29^. Figure 1 shows an overlap image of lesion tracings manually registered to the Montreal Neurological Institute (MNI) brain by the same neurologist. Demographic information and lesion volumes are provided in Table 1, and neuropsychological screening results in Table 2. Performance on attention and executive function tests, and the frequency of use of psychoactive medications, was comparable across patient groups. The DMF group scored lower than the VMF group on a test of incidental memory, and both VMF and DMF groups scored higher than controls on the Beck Depression Inventory-II.

Subjects viewed a set of 175 artworks, one at a time, judging how much they wanted each artwork on a scale of −3 to 3 (‘not at all' to ‘very much'). A subset of artworks drawn from those rated zero or above were then paired and presented in a binary choice task, similar to Krajbich *et al*.^3^, with an equal number of trials at each level of rating difference (that is, 0, 1, 2), while eye movements were tracked. Subjects also rated the brightness of a separate set of 50 artworks on the same scale in a control task. The consistency of ratings was assessed by having subjects re-rate the brightness of these artworks, and the value of 50 artworks from the initial set, not shown in the choice task, at the end of the session.

### Reaction times and fixation properties

**Figure 2:**
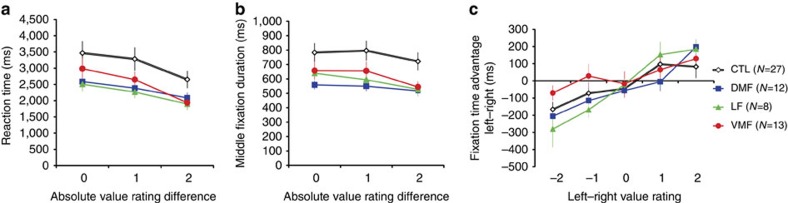
Reaction times and fixation properties for the choice task. (**a**) Mean reaction time as a function of the absolute value rating difference of the two options. (**b**) Mean number of fixation shifts between options per trial as a function of the absolute value rating difference of the two options. (**c**) Mean fixation time advantage to the left versus the right option as a function of the value rating difference of the left and right options. White lines show data from control subjects (CTL), blue lines show data from DMF group, green lines show data from LF group and red lines show data from VMF group. Error bars indicate s.e.m.

We first asked whether PFC damage affected basic aspects of choice task behaviour, focusing on the effects of value rating difference on choice reaction time (RT) and eye movements. As the value difference between the two options increased, patients and controls made faster decisions (Mixed measures analysis of variance (ANOVA): *F*_2,112_=25.89, *P*<0.0001) (Fig. 2a). There was no significant main effect of group (*F*_3,56_=2.05, *P*=0.1), or interaction between group and rating difference (*F*_2,112_=0.75 *P*=0.6). The same pattern was seen after removing RT outliers (Supplementary Fig. 1).

In describing eye movements in this task, we use the term ‘fixation,' as defined in prior work^3^,^4^, to refer to a set of eye movements towards one option before eye position shifted to the opposite option. Subjects shifted fixation between options less often as the value difference between the options increased (Supplementary Fig. 2). We also examined the average fixation duration between the first and last fixation (‘middle fixation duration') during the choice task. Middle fixation duration was shorter as the value rating difference increased (Mixed measures ANOVA: *F*_2,112_=16.57, *P*<0.0001), as expected^3^. There was a significant main effect of group (*F*_2,56_=2.95, *P*=0.04), but no interaction between group and value difference on middle fixation duration (Fig. 2b; *F*_6,112_=1.02, *P*=0.4). *Post hoc* tests collapsed across value differences showed that middle fixation duration was significantly shorter in all the PFC groups compared with controls (Bonferroni corrected *t*-tests: *P*'s≤0.02, two-tailed).

Subjects spent more time fixating highly rated options (Fig. 2c), with a significant effect of the value rating difference between the left and right option on fixation advantage (time spent fixating left–right option; mixed-measures ANOVA: *F*_4,224_=15.45, *P*<0.0001). This tendency was comparable in all the groups, with no interaction between value difference and group (*F*_4,224_=0.87, *P*=0.6), or main effect of group (*F*_3,56_=1.03, *P*=0.4).

### Analysis of choices

We next asked whether PFC damage changed the relative influence of a priori value ratings and ‘in the moment' evaluation indexed by fixation time. Generalized estimating equations (GEEs) were used to predict choice of the left option as a binary outcome based on the rating difference of the left and right options, and whether the option was looked at longer (fixation advantage), on individual trials. Rating differences ranged from −2 to 2 (lower to higher rating for left option), while fixation advantage was divided into five bins. Recent work has also shown that visual saliency can bias choices, independent of value^6^,^30^,^31^. We therefore included a binary variable coding which option had greater visual saliency.

Prior work with this task has tested effects of fixation advantage on choice without accounting for the influence of value ratings and saliency on fixations^3^,^4^. Both saliency and value can influence fixation times^6^, posing difficulties for disentangling the effects of these variables. To isolate the information-gathering process that was the focus of this study, we calculated a ‘predicted fixation advantage' based on the a priori value rating difference and binary saliency variable for each subject, in each trial. This value was subtracted from subjects' actual fixation advantage, leaving residuals reflecting the fixation advantage not predicted by differences in value ratings and saliency. This variable was used in all further analyses. For consistency with the prior literature, the data were also analysed using the raw fixation advantage, yielding a very similar pattern of results (Supplementary Fig. 3).

Starting with a simple model that only included main effects, we systematically added interactions between group status and each variable. The optimal model was selected based on the minimum Quasi-Akaike Information Criterion (QIC). The tested models and associated QIC statistics are provided in Supplementary Table 1. Odds ratios (ORs) and 95% confidence intervals (CIs) are reported for each effect, reflecting the change in probability of choosing the left option as a function of each variable for main effects, and the relative change in this probability in PFC groups compared with controls in the interactions.

**Figure 3:**
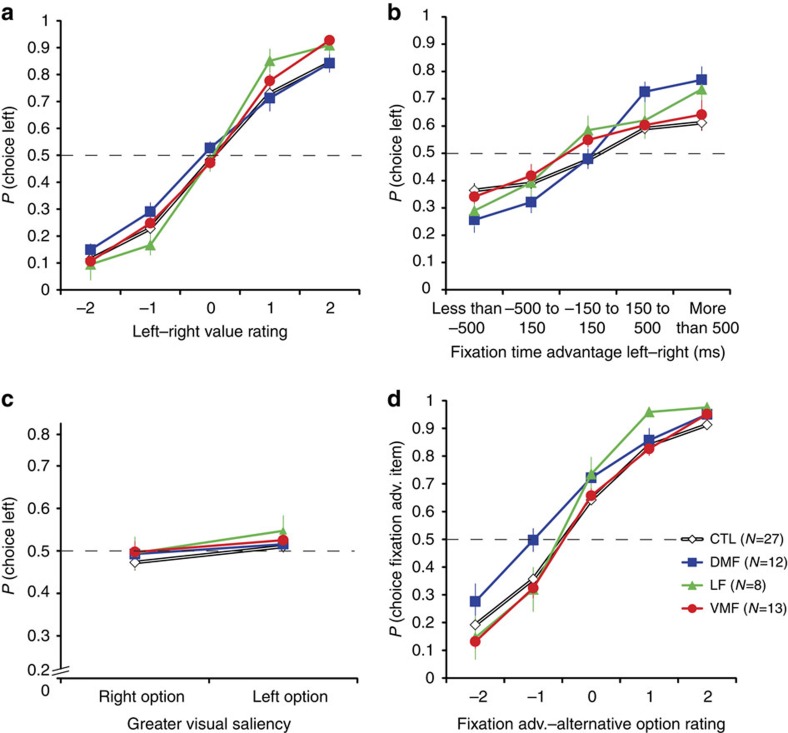
Choice properties of each group. (**a**) Probability of choosing the left option as a function of the value rating difference of the left and right option. (**b**) Probability of choosing the left option as a function of fixation advantage (adv.) to the left versus the right option (**c**) Probability of choosing the left option as a function of the difference of the visual saliency of the left and right options. (**d**) Probability of choosing the option with a greater fixation advantage as a function of the value rating difference of the fixation-advantaged and the alternative option. Error bars represent the s.e.m. Dashed line indicates chance probability.

The optimal model included interactions of group with rating difference and fixation bias, but not saliency (Fig. 3a–c). We found significant main effects of rating difference (OR: 2.79, CI: 2.45–3.19, *P*<0.0001), fixation advantage (OR: 1.47, CI: 1.37–1.59 *P*<0.0001) and saliency (OR: 1.16, CI: 1.02–1.32, *P*=0.02) on choice, replicating previous work^3^,^6^. There were no significant main effects of group (DMF: OR: 1.22, CI: 0.96–1.54, *P*=0.1; LF: OR: 1.07, CI: 0.74–1.54, *P*=0.7; VMF: OR: 1.12, CI: 0.92–1.38, *P*=0.2).

To test whether patients made choices that were as consistent with their value ratings as the control group, we examined the interaction of group with value rating difference (Fig. 3a). There was no significant effect for the DMF group (OR: 0.99, CI: 0.78–1.25, *P*=0.9). However, LF (OR: 1.65, CI: 1.04–2.63, *P*=0.03) and VMF (OR: 1.24, CI: 1.00–1.53, *P*=0.05) groups both made choices that were slightly more consistent with their ratings compared with controls.

We also examined the interaction of group with fixation advantage to test whether PFC damage affected the influence of fixations on choice (Fig. 3b). Fixation advantage has a stronger influence on choice in the DMF group compared with controls (OR: 1.54, CI: 1.18–2.00, *P*=0.001). In contrast, the effects of fixation advantage on choice in the LF (OR: 1.41, CI: 0.97–2.03, *P*=0.07) and VMF (OR: 1.09, CI: 0.93–1.28, *P*=0.3) groups were not significantly different from controls.

One key prediction of previous work with this paradigm is that an increased influence of fixations should be accompanied by decreased sensitivity to the value of the unfixated option^3^. To address this, choices of the fixation-advantaged option were modelled in a separate GEE as a function of the value rating difference of this option and the alternative, with the magnitude of fixation advantage and saliency included as nuisance variables (Fig. 3d). Including the interaction of group with rating difference improved the fit over the simple model without interactions (Supplementary Table 1). The DMF group was overall more likely to choose the fixation-advantaged option (OR: 1.49, CI: 1.13–1.95, *P*=0.004), while VMF (OR: 0.98, CI: 0.81–1.21, *P=*0.9) and LF (OR: 1.17, CI: 0.75–1.83, *P=*0.5) groups were not significantly different from controls. There was a significant main effect of the value rating difference between the fixation-advantaged and alternative option (OR: 3.15, CI: 2.80–3.55, *P*<0.0001), as expected. However, the DMF group was less sensitive to this value rating difference (OR: 0.75, CI: 0.65–0.88, *P*=0.0003), while VMF (OR: 1.02, CI: 0.90–1.16, *P=*0.7) and LF (OR: 1.02, CI: 0.73–1.44, *P=*0.9) groups did not differ from controls. There were also significant main effects of saliency (OR: 1.15, CI: 1.01–1.31, *P*=0.03), and fixation advantage magnitude (OR: 1.42, CI: 1.28–1.57, *P*<0.0001). The DMF group's choices were therefore biased toward the fixation-advantaged option, and less sensitive to the value difference between this option and the alternative.

We explored whether patients responded differently to options presented in the contra- or ipsilesional hemifield in the 25 patients with unilateral damage. There were no differences in the effects of fixation advantage, or in the likelihood of subjects choosing the option with a higher value rating for contra- or ipsilesional options, nor did any group preferentially fixate options in either hemifield (Supplementary Figs 4 and 5).

### Attentional drift diffusion model

One advantage of this paradigm is that a formal model has been developed that captures several features of the effect of fixations on choice (attentional drift-diffusion model (aDDM)^3^). In this framework, decisions are modelled as a diffusion process that progresses at a rate dependent on the value difference of available options. Critically, in the aDDM, this rate is weighted by a parameter that discounts the value of the unfixated option, resulting in a bias toward choosing the fixated option. In an exploratory analysis, we fit the model to individual subject data to examine whether the effects of prefrontal damage in this task could be captured by changes in the parameters of this model.

**Figure 4:**
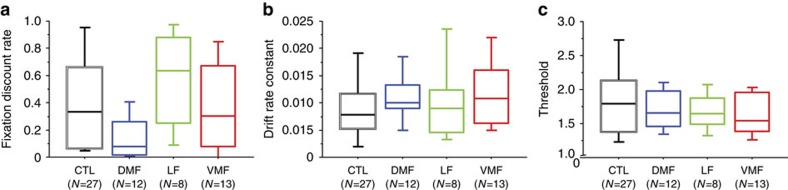
Parameter estimates from the attentional drift diffusion model (aDDM) in each group. (**a**) Fixation discount rate. (**b**) Drift rate constant. (**c**) Threshold parameter. Box plots show the 10th, 25th, 50th, 75th and 90th percentiles.

The model included three free parameters: the fixation discount rate, a drift constant that controlled the rate of drift, and a threshold that determined the necessary height of the drift process to trigger a choice (see Methods). There was a significant effect of group on the fixation discount rate (Fig. 4a; Kruskal–Wallis test: *H*_3_=8.11, *P*=0.04), driven by a lower fixation discount rate in the DMF group. *Post hoc* tests showed a trend toward a difference between the DMF and LF groups (Bonferroni corrected Mann–Whitney *U*-test: *Z*=2.55, *P*=0.07, two-tailed), and controls (*Z*=2.40, *P*=0.1), and no other notable differences (*Z*≤1.36, *P*⩾0.9). There were no significant group effects on drift rate constant (Fig. 4b; Kruskal–Wallis test: *H*_3_=2.33, *P*=0.5) or threshold (Fig. 4c; Kruskal–Wallis test: *H*_3_=1.51, *P*=0.7). There were also no differences between groups in the fit of the model (Supplementary Fig. 6).

### Rating consistency

Activity within VMF has been shown to scale with relative value in similar tasks studied using fMRI^11^,^12^,^28^, but to our knowledge, there has been no direct test of whether any PFC region is necessary for consistently assigning a value rating to a stimulus. In a secondary analysis, we asked whether value ratings were more inconsistent in any frontal group by comparing the absolute difference and correlation of ratings of a separate set of artworks before and after the choice task.

**Figure 5:**
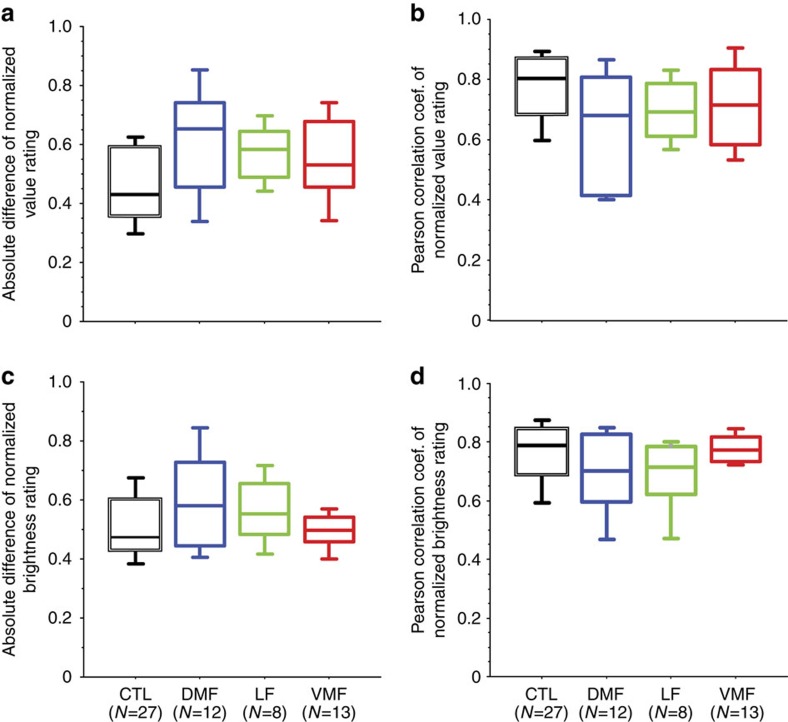
Measurements of consistency of value and brightness ratings of artworks in the test and retest phase of experiment by group. (**a**) Absolute difference of normalized value ratings. (**b**) Pearson correlation coefficients (coef.) of normalized value ratings. (**c**) Absolute difference of normalized brightness ratings. (**d**) Pearson correlation coefficients of normalized brightness ratings. Box plots show the 10th, 25th, 50th, 75th and 90th percentiles.

There was a marginally significant effect of group on absolute value rating difference (between-subjects Kruskal–Wallis test: *H*_3_=7.74, *P=*0.05; Fig. 5a). The DMF group was numerically more inconsistent, however, *post hoc* tests between groups did not find significant differences (Bonferroni corrected Mann–Whitney *U*-tests: *Z*≤2.22, *P*⩾0.16, two-tailed). When consistency was assessed by the correlation between test and retest ratings, there was no significant effect of group (between-subjects Kruskal–Wallis test: *H*_3_=5.84, *P=*0.1; Fig. 5b).

There was no significant effect of group on the absolute brightness rating difference (between-subjects Kruskal–Wallis test: *H*_3_=3.14, *P=*0.4), nor any effect of group on the correlation between test and retest brightness ratings (between-subjects Kruskal–Wallis test: *H*_3_=5.72, *P=*0.1; Fig. 5c,d).

We tested the relationship of value rating inconsistency with a simple index of fixation bias (Supplementary Fig. 7) within control subjects. Simple regression analyses showed that fixation bias was correlated with age and education. We therefore included them in a multiple linear regression model as nuisance variables. Age and education were significant predictors of fixation bias, whereas value rating consistency was not (Table 3). Fixation bias residuals for all the subjects were calculated on the basis of the coefficients of this regression analysis. The increased fixation bias in the DMF group survived this correction, and was therefore not a result of inconsistent value ratings, or of age or education (Supplementary Fig. 8). Worse incidental memory performance in the PFC group as a whole showed a weak relationship with inconsistent value ratings, but not the degree of fixation bias (Supplementary Fig. 9).

### Voxel-based lesion symptom mapping

**Figure 6:**
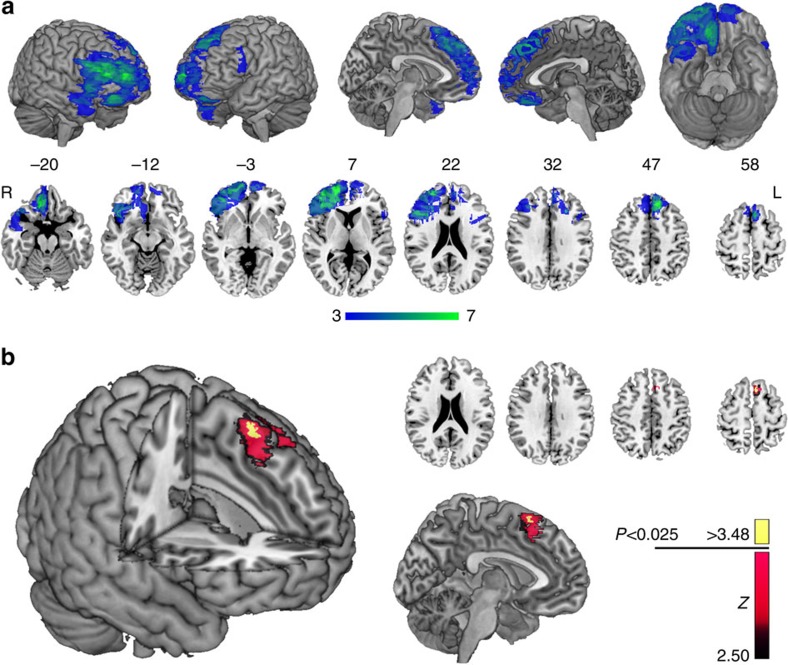
Voxel-based lesion symptom mapping (VLSM) for the effect of fixation advantage on choice. (**a**) Map showing the voxels where there was sufficient lesion overlap to detect an effect using VLSM methods, overlaid on the MNI brain in three-dimensional views, and in axial slices. Numbers above the axial slices correspond to *z* coordinates in MNI space. The colour scale indicates the number of patients with lesion overlap in any given voxel. L, left; R, right. (**b**) VLSM statistical map computed for the effect of fixation advantage on choice overlaid on the MNI brain in a three-dimensional view (left), as well as a mid-sagittal view showing the medial wall of the left hemisphere (bottom) and on representative axial slices (top). The colour scale indicates Brunner–Munzel *Z* scores. Voxels in yellow indicate where this effect was significant at *P*<0.025, corrected with permutation tests.

Although the region-of-interest approach provides evidence for a necessary role of DMF in mediating fixation bias, this method artificially limits regional specificity. Voxel-based lesion symptom mapping (VLSM) can overcome this limitation by testing the impact of damage on behaviour at the voxel level^32^,^33^. VLSM is constrained by the degree of lesion overlap in the sample as a whole. In keeping with standard practice, we included voxels that were damaged in three or more patients in this analysis^34^,^35^,^36^. Figure 6a shows the voxels where lesion–function relationships could be tested with this method in this study.

The residualized fixation bias index was entered into the VLSM analysis. The nonparametric Brunner–Munzel test^37^ was applied at all voxels with sufficient lesion overlap, and the threshold for statistical significance was determined using permutation testing to correct for multiple comparisons. Figure 6b shows voxels where damage was associated with an increased effect of fixation advantage on choice. Damage involving the left superior frontal gyrus was most strongly related to the behavioural effect. The strongest statistical effect (*P*<0.025) was in a small cluster of voxels (MNI: −7, 20, 61) in the rostral pre-supplementary motor area (pre-SMA)^38^,^39^.

### Correlation of artwork ratings

**Figure 7:**
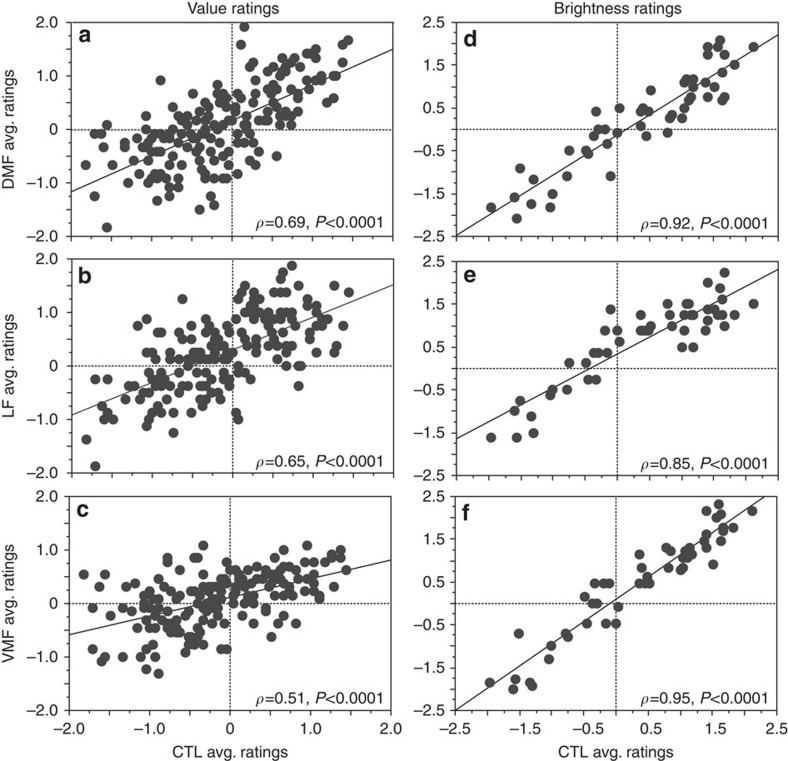
Scatterplots of average ratings of all artworks for each group in the value rating task (175 artworks) and brightness rating task (50 artworks). (**a**–**c**) Average (avg.) artwork value ratings of (**a**) DMF, (**b**) LF and (**c**) VMF groups are plotted against control subjects (CTL). (**d**–**f**) Average artwork brightness ratings for (**d**) DMF, (**e**) LF and (**f**) VMF groups are plotted against the control group. Average only includes subjects' initial ratings (that is, no data from the retest phase). Spearman correlation coefficient and associated *P* values are shown in the bottom right-hand corner of each panel.

Somewhat to our surprise, VMF patients made consistent value ratings, and choices consistent with these values. We explored whether the value judgments of these patients were similar to those of controls. The control subjects' average value ratings for each artwork were significantly correlated with the average value ratings of all the three PFC groups, but this relationship was weaker in the VMF group (Fig. 7a–c). In particular, the VMF group's average ratings were more variable for artworks that the control group had rated below ‘0.' In contrast, ratings of artwork brightness were highly correlated between controls and all the PFC groups (Fig. 7d–f).

## Discussion

Several recent studies have found that visual fixations during deliberation can influence value-based decisions^3^,^4^,^5^,^6^,^7^,^8^, an observation that opens a novel window on the dynamic construction of value during choices. Here, we applied this experimental approach to test whether focal PFC damage affects this aspect of value updating. The choices of all the groups were influenced by both their a priori value assessment of the options, and by fixations during the choice process. Damage to DMF, and not other PFC regions, was associated with a significant increase in the influence of fixations on choice.

Existing efforts to relate fixation-driven updating to neural mechanisms have focused on value-related signals in ventromedial PFC^28^. That account would predict a reduced influence of fixations on choice after VMF damage, which we did not observe. Indeed, the VMF group was intact in nearly all aspects of this task, a striking finding given the putative central role of this region in current models of value-based decision-making^10^,^12^,^18^. Previous work from our group has found that VMF damage disrupts preference transitivity^14^,^15^. This finding has been taken as evidence that this region is required for consistent comparisons, but could support other models of the role of this region, such as a role in constructing a superordinate hierarchy of option values^40^. Here, for the first time, we directly tested the stability of value ratings for individual options, and found that VMF-damaged patients provided consistent ratings over the course of the experiment and made choices that were more consistent with these ratings (in economic terms, more rational) than controls. These findings align with studies in monkeys demonstrating intact preference-based choice after orbitofrontal lesions^41^, and present a challenge to simple views of ventromedial PFC as universally necessary for assessing and comparing the economic values of options.

The intact, even supranormal, behaviour of VMF-damaged patients might reflect differences in the information used to make value ratings. As with all ecologically valid stimuli, artwork can be assessed on a range of attributes^42^, presumably integrated to produce a single subjective value estimate that has been a focus of much neuroeconomics work to date. Imaging studies suggest a role for VMF in the integration of value information from multiple sources^26^,^27^. Here, value ratings of the VMF group agreed less with those of controls, suggesting these assessments might be based on different attributes, or different attribute weightings. In recent work with social stimuli, we found that VMF damage disrupts the integration of attributes: these patients used simpler information to inform their choices than did controls^43^. Thus, VMF damage may affect the information used by patients to assign value to items, rather than the ability to report or compare those values once assigned. Indeed, subjects with VMF damage may simplify the value construction problem as a compensatory strategy^44^. Addressing this possibility fully will require approaches that impose better experimental control over the ‘value construction' process that work in this field to date has largely left unconstrained.

Damage to DMF, and not other PFC regions, was associated with an increase in the influence of fixations on choice. An exploratory model-based analysis of this data set suggested that the increased influence of fixations in the DMF group could be accounted for by discounting the value of the unfixated option during the decision process. Although the results of this *post hoc* model-based analysis should be considered preliminary, they complement the main GEE analyses that took full advantage of this data set. Those primary analyses showed a robust increase in the influence of fixations in the DMF group and decreased sensitivity to the value rating difference of the fixation advantaged and alternative options, as would be predicted by a decrease in the fixation discount rate in the model^3^. DMF may thus be necessary for maintaining the value of unattended options. This result aligns with findings from foraging tasks where activity within DMF tracked the value of departing from a default option and exploring alternatives^19^,^20^. The fMRI data supporting this view have recently been challenged with the alternative hypothesis that this signal reflects choice difficulty or conflict^45^. The predictions of the latter model in terms of lesion effects in this task are not entirely clear, but we note that our prior work has failed to find consistent effects of DMF lesions on behavioural indices of conflict monitoring in a variety of tasks^46^. Further, here we found that the performance of DMF patients was sensitive to choice difficulty, as indexed by RT and fixation data.

These findings agree with other work placing DMF at a critical juncture linking value comparison and action selection^21^,^22^,^47^. Voxel-based analysis in this large PFC-damaged sample revealed that increased fixation bias was driven by damage within the pre-SMA. Damage to the pre-SMA/SMA has been associated with failure of goal-directed control over externally triggered responses^48^,^49^, and can cause utilization behaviour, a clinical phenomenon in which behaviour is excessively influenced by environmental cues (see Iaccarino *et al*.^50^ for a recent review). The increased fixation bias associated with pre-SMA damage in the current study could be interpreted as exaggerated environmental control over decision-making, arguing for a specific role of pre-SMA in mediating the influence of attention during the choice process.

Human lesion studies have intrinsic limitations that should be kept in mind in interpreting these results. Sample size is limited by practical considerations, particularly when the behaviour is measured in detail, as here. The power to detect effects also varies with the extent of lesion overlap and the covariance of damage within individual lesions. Although the sample studied here is relatively large by the standards of such work, and the expected effect size in lesion studies is moderate-to-large, power remains a perennial concern in interpreting both the null and positive findings^51^. The overlap map (Fig. 6a) is a guide to those PFC regions we are best placed to test in this sample. It should be noted that these lesions also affect the underlying white matter to varying degree. Although consistent effects of white matter damage should be revealed in the voxel-based analysis, it is difficult to fully distinguish effects of cortical damage from effects on underlying fibers of passage with potential impact on brain function at a distance from the site of injury. The findings thus need to be considered in the context of evidence from multiple techniques.

Finally, we note that the design of the experiment required that initial ratings meet certain criteria. Two VMF subjects and one healthy control were excluded from the experiment for failing to meet these criteria, biasing the VMF sample towards those able to make value ratings along the specified range.

These findings provide convergent support for some, but not all, elements of current neurobiological models of the influence of attention on value-based choice. We clearly show that intact VMF is not required for fixation-based value updating, challenging a simple view of VMF as a general, dynamic ‘value-meter'. In contrast, DMF makes a necessary contribution to this process, allowing information about the value of the currently unattended option to influence choice.

## Methods

### Subjects

Patients with focal lesions involving the frontal lobes (*N*=33) were recruited from the Cognitive Neuroscience Research Registry at McGill University^52^. They were eligible if they had a fixed lesion primarily affecting the frontal lobes. They were tested a minimum of 5 months after the injury (median, 4.76 years after; range: 5 months to 48 years). Twelve patients were taking psychoactive medications: eight were taking anticonvulsants, five were taking antidepressants and two were taking anxiolytics. There was no significant difference in the frequency of psychoactive medication use between PFC groups (Chi-square test of independence: *χ*^2^=2.65 (2), *P=*0.3). A power analysis of pilot data collected from healthy young subjects was used to inform the determination of group sample size.

Age- and education-matched healthy control subjects (*N*=27) were recruited through local advertisement in Montreal. They were free of neurological or psychiatric disease and were not taking any psychoactive drugs. They were excluded if they scored 26 or less on the Montreal Cognitive Assessment^53^. Mean performance was 28.1, s.d.=1.4.

Two subjects (one control and one VMF-damaged patient) were excluded from the study because they did not rate enough artworks above ‘0' to generate trials for the choice task. Another VMF patient was excluded because she only used ‘0' and the extremes of the value scale (‘−3' and ‘3'), making it impossible to generate trials with the same value rating differences as the other subjects. One DMF patient was excluded because there was residual tumour evident on imaging, so that the extent of the lesion could not be characterized with confidence. One patient with LF damage was excluded as she failed to understand the task instructions.

All the subjects provided written, informed consent in accordance with the Declaration of Helsinki and were paid a nominal fee for their time. The study protocol was approved by the McGill University Research Ethics Board.

### Lesion analysis

Individual lesions were traced from the most recent clinical computed tomography or magnetic resonance imaging onto the standard MNI brain using MRIcro software^54^ (freely available at www.mccauslandcenter.sc.edu/mricro/) by a neurologist experienced in imaging analysis and blind to task performance. A related software tool (MRIcron) was used to generate lesion overlap images and estimate lesion volumes. Patients were separated into groups on the basis of the location of damage by the same neurologist. The grouping of subjects conformed to broad divisions of the PFC used in neuropsychological studies of PFC damage^29^,^55^. One patient in the DMF group had a second lesion in the parietal lobe. This patient's fixation bias was not driving the group effect (normalized fixation bias: 0.10). Another patient in the VMF group also had damage in the parietal white matter, but again, this patient's behaviour was not notably different from the rest of the VMF group (normalized fixation bias: −1.17). DMF lesions were owing to tumour resection in 10 cases, aneurysm rupture in one case and haemorrhagic stroke in one case. Lesions in the LF group were caused by ischemic stroke in five cases and tumour resection in three cases. Lesions affecting VMF were attributable to tumour resection in nine cases, aneurysm rupture in three cases and haemorrhagic stroke in one case.

### Neuropsychological screening

All the patients underwent neuropsychological screening to assess cognitive functions more generally, to detect deficits that might affect experimental task performance for other reasons. Hemispatial neglect was tested with the Posner cueing task^56^, and a circle cancellation task^57^. Patients also completed a task that tested visual memory for faces without explicit instructions (incidental memory)^58^, two tests of verbal fluency, a well-established index of left frontal function (Fluency-F, Animals)^59^, a test of working memory (backwards digit span)^60^ and a test of the ability to understand and follow one, two and three-step verbal instructions (sentence comprehension, similar to the Token Test^61^).

### Apparatus

All the experimental tests were programmed using E-Prime 1.2 (Psychology Software Tools, Inc., Pittsburgh, PA, USA). Subjects' heads were stabilized using a headrest and stimuli were presented on a 19-inch monitor (Dell Inc., Round Rock, TX, USA) positioned ∼57 cm from their eyes. Monocular recordings of the movement of each subject's dominant eye were acquired at 1,000 Hz using an Eyelink 1000 system with a desk-mounted camera (SR Research Ltd., Mississauga, Ontario, Canada).

### Rating tasks

Subjects completed two separate rating tasks, one where they were asked to judge how much they wanted the presented artwork, and a second control task where they were asked to judge the brightness of a separate set of artwork. The artwork was sampled from a wide range of styles and periods, and included pieces from both famous and lesser-known artists. The diversity of artworks presented to subjects was intended to encompass the idiosyncrasies of individual preferences. In both tasks, we tested the consistency of subjects' responses by asking subjects to judge a subset of the artwork again for both value and brightness at the end of the testing session. The purpose of this retest phase was to establish whether patients' rating of the value of artwork was stable over time, and to determine whether any inconsistency was specific to value ratings.

Subjects were first asked to rate how much they wanted to have 175 individual pieces of artwork on a scale of −3 to 3. On each trial, a central fixation cross was presented for 500 ms. Subjects would then see the artwork in the centre of the screen, as well as a prompt above-the-artwork reading ‘How much do you want this artwork?' The scale was presented below the artwork, with labels below −3 (‘Not at all'), 0 (‘Indifferent') and 3 (‘Very much.') Subjects would verbally report a number to the experimenter, who would then click the corresponding number using a computer mouse. The first 125 artworks presented to subjects in the rating task were used to generate pairs of artwork for the choice task (see below). The remaining 50 artworks were presented to subjects again after the choice task in the retest phase. The order of artwork presentation was randomized for every subject.

After the first rating task, subjects were asked to judge the brightness of a separate set of 50 artworks. These artworks covered the same wide range of subject matter and style as the set in the first test, and also varied considerably in mean luminance. The format of this task was nearly identical to the first rating task. In each trial, subjects were presented with a central artwork, and a prompt reading ‘How bright is this artwork?' Subjects rated each artwork on a scale of −3 to 3 by reporting a number to the experimenter. Labels appeared below −3 (‘Very dull'), 0 (‘Neutral') and 3 (‘Very bright.') Subjects were again asked to judge the brightness of these same 50 artworks at the end of the experiment during the retest phase. All the subjects reviewed the instructions for each task with the experimenter before starting each test. In the brightness rating task, subjects were specifically instructed to rate artwork for perceptual brightness rather than mood of the artwork.

### Choice task

The design of the choice task was very similar to that used by Krajbich *et al*.^3^ A custom-made Matlab (Mathworks, Natick, MA, USA) script sorted the first 125 artworks presented to subjects in the value rating task into pairs for the choice task. Similar to Krajbich *et al*.^3^, any artworks that subjects had rated below ‘0' (that is, artworks they did not want) were excluded from the choice task. The script selected pairs of artworks on the basis of the difference in subjects' ratings, with three levels (0, 1 or 2). There were 34 pairs for each difference level in the choice task (102 pairs total). This script also ensured that no artwork appeared more than eight times during the course of the task.

The subjects were instructed to choose which artwork they wanted more from each of the presented pairs. The subjects were told that they would receive a copy of one of the artworks they chose at the end of the experiment to provide an incentive for answering honestly. The subjects received a postcard-sized copy of the artwork they chose in the final trial of this task. This trial was not included in any analysis.

All the subjects completed a 13-point eye-tracker calibration sequence covering a 32.1 by 26.6° area before beginning the task. On each trial, subjects had to hold fixation on a central fixation cross for 500 ms before the trial began. This process also served to ensure the quality of calibration throughout the test: Failure to maintain fixation in a 1.6 by 1.8° box around the fixation cross would cause the fixation slide to repeat. After three consecutive failures, the eye-tracker would be recalibrated. After holding central fixation, subjects were presented with two artworks on either side of the screen (the side of the artworks was randomized with respect to their ratings). The subjects were allowed to freely inspect the artwork for an indefinite period before finally making a choice by pressing the left- or right-most keys of a serial response box (Psychology Software Tools, Inc.) to choose the artwork on the corresponding side. After making a choice, the selected artwork was highlighted with a yellow border for 1,000 ms. The subjects then saw a blank screen for 1,000 ms before the start of the next trial.

### Eye-tracking analysis

Fixations were defined using the online parser of the Eyelink 1000: saccades were identified using a velocity threshold of 30° per second, an acceleration threshold of 8,000° per second squared and a distance threshold of more than 0.15°. This same parser also automatically rejected blinks. In-house written Matlab (Mathworks) scripts were used to determine the location of fixations and extract the data for analysis. Trials where subjects did not make a fixation to either option were rejected from further analysis (0.21% of all trials).

### Choice task analysis

Fixations were defined as a set of continuous eye movements made to either option, and fixation shifts were defined as fixations where the subject shifted eye position from one option to the other. The number of fixation shifts measured how many times subjects broke their fixation from one option to look at another during the course of a trial. The middle fixation time was calculated as the average duration of all the fixations that fell between the first fixation and the last fixation on any given trial, as in Krajbich *et al*.^3^ This usage of the term ‘fixation' is consistent with prior studies using this paradigm^3^,^4^, though it is somewhat different from the definition typically used in the eye-movement literature.

Fixation time advantage was calculated by taking the difference of the total time subjects spent fixating the left and right option by the end of the trial (at the point where the subject made a choice). To assess how fixation advantage influenced choice, trials were binned based on this measurement. The size of these bins was set to ensure that trials were relatively well distributed among bins (mean number of trials in each bin: less than −500 ms (*M*=17.0, s.d.=9.0), −500 to −150 ms (*M*=20.9, s.d.=6.6), −150 to 150 ms (*M*=26.1, s.d.=10.0), 150 to 500 ms (*M*=20.0, s.d.=5.7), more than 500 ms (*M*=17.8, s.d.=8.8)).

### Saliency analysis

Saliency maps were calculated for all the artworks used in the choice task using the SaliencyToolbox, an open-access Matlab (Mathworks) tool for evaluating the saliency of computer images based on simple visual features^62^. The default toolbox parameters were used, where colour, orientation and intensities were all equally weighted (weight=1.0). The sum of the saliency maps were computed for each image as in Towal *et al*.^6^ As this measure was negatively correlated with the area of the image (nonparametric Spearman rho correlation: *ρ*=−0.437, *P*<0.0001), we corrected each saliency estimate for the area of the image based on this linear correlation to obtain a more accurate estimate. Trials were then classified on the basis of whether the estimated visual saliency of the left or right option was higher.

### Comparison of effects between hemifields

The effects of fixation advantage and value ratings were compared for options presented in the contra- or ipsilesional hemifields. This analysis could only be completed in 25 patients where damage was restricted to one hemisphere. The influence of value ratings in each hemifield was tested by comparing the frequency subjects chose options with a higher value rating when presented contra- or ipsilesionally. Similarly, the effect of fixation advantage was tested by comparing the frequency subjects chose the option fixated for longer when presented in the contra- or ipsilesional hemifield. This measure was corrected for the value rating difference of the options by subtracting the frequency that subjects chose the fixation-advantaged option, given their value rating difference, from subjects' choice of the fixation-advantaged option (0 or 1) in each trial, as in Krajbich *et al*.^3^

### Attentional drift-diffusion model

The aDDM was fit to individual subject data to test whether the effects of prefrontal damage on task performance could be systematically related to changes in the parameters of this model. This model was originally described by Krajbich *et al*.^3^, and is based on the drift-diffusion model developed by Ratcliff^63^. In the aDDM, binary choices are modelled as a stochastic diffusion process moving between two equidistant barriers reflecting the instantaneous relative decision value (RDV). When the process crosses the barrier set by the threshold, a decision is made. A unique feature of this model is that the direction of this process depends on the locus of fixation, such that when fixations are made to the left, the diffusion process changes at every time point according to *RDV*_*t*_*=RDV*_*t*-1_+*d*(*V*_left_−*θV*_right_)+*s*, and when fixations are made to the right according to *RDV*_*t*_*=RDV*_*t*-1_−*d*(*V*_right_−*θV*_left_)+*s*. Here, *V*_left_ and *V*_right_ represent the value ratings of the left and right options, respectively, and the parameter *θ* represents the fixation discount rate on the range of 0 to 1. The parameter *d* is the drift constant, governing the rate of integration. The parameter *s* represents the variability in the drift rate and acts as a scaling parameter. Here, *s* was set to a constant (0.1) multiplied by Gaussian white noise randomly sampled every time step.

Within the model, the RDV was sampled every 10 ms in simulated trials based on the equations described above. In each simulated trial, the location of the first fixation was based empirically on the frequency the subject looked left or right first in all trials. The duration of each fixation was randomly sampled from the maximum-likelihood estimate of the lognormal distribution of the subject's fixation times to the side of fixation at each level of value difference for the left and right options (−2, −1, 0, 1, 2). Model reaction times were computed from the time the RDV crossed the threshold, plus the ‘non-decision time,' calculated from the empirical mean time to the first fixation. As each fixation is considered instantaneous in the model, a transition time was added to the RT for each simulated fixation. Transition times were randomly sampled from the maximum-likelihood estimate of the lognormal distribution of subjects' empirical transition times.

We fit the model to all trials for each subject. While this approach risks over-fitting the model to the data and prohibits cross-validation, it was necessary to allow even an exploratory analysis, given the small number of trials in the experiment. For each simulation, 12,000 trials were generated for each subject. The composition of trial conditions was directly based on the proportion of trials in each condition in each subject's data (that is, same proportion of trials where the left option was rated ‘3' and the right option was rated ‘1' and so on).

The model was fit using Kolmogorov–Smirnov equations, based on the method of Voss, Rothermund^64^. Subject and simulated data were split into three conditions based on the absolute value difference of the options (0, 1, 2). Given the low number of trials, we collapsed across left and right side, as we did not find any systematic bias towards the choice of a particular side in any group. All the reaction times were included in a single distribution for each condition; with choices of the low value option assigned a negative sign (for trials with a value difference of zero, choices of the left option were arbitrarily negative signed). Outlier RTs (outside the 0.005 and 0.995 quantiles of the RT distribution) were removed from the subject data and simulated trials to improve the fit of the model. For each condition, the fit of the simulation to the subject's data was assessed using the Kolmogorov–Smirnov test. The objective function for the model fitting procedure was the sum of the negative natural logarithm of the *P* values from these three tests. Model parameters were fit using the pattern search algorithm in the Matlab global optimization toolbox (Mathworks). The theta parameter was constrained to the range of 0 to 1, the drift constant to a range of 0.001 to 0.05, and the threshold parameter to a range of 0.75 to 4.0. For each subject, the model fitting procedure was run 10 times, nine times at random initialization points and once from a fixed, centred point. The set of parameters that best minimized the objective function were then selected for each subject.

### Rating consistency analysis

To adjust for individual differences in rating anchoring, and in the range of the scale utilized, the brightness and value ratings of artworks used in the test–retest phases were normalized. Ratings were converted into *Z*-scores based on the means and standard deviations of subjects' ratings of these options in each phase. The mean absolute difference of the normalized ratings of these artworks was then calculated for each subject to measure rating consistency.

The relationship between fixation bias and value rating consistency was tested in healthy controls using a multiple linear regression model that incorporated age and education as nuisance variables, as they were found to correlate with fixation bias in simple regression analyses. We computed a single index of fixation bias based on the difference in the probability that subjects chose the left option when they spent more time fixating the left or right option. To remove the influence of the value difference of options on choice, we corrected subjects' choices of the left option (0 or 1) by subtracting the average frequency the subject chose the left option given the value rating difference of the options in each trial as in Krajbich *et al*.^3^ The fixation bias index was calculated from the difference in this corrected probability of choosing the left option for trials where there was a greater fixation advantage to the left or the right. Control subjects' fixation bias was converted to a *Z*-score based on the mean and standard deviation of the group to standardize the coefficients. Education was incorporated as an ordinal variable with three levels (high school or less, some undergraduate education to undergraduate degree, some graduate education to graduate degree).

To determine whether differences between groups were an artifact of age, education or value-rating inconsistency, we calculated the residuals of fixation bias not accounted for by these variables in all the subjects. The fixation bias index was calculated for all the subjects and normalized with reference to the mean and standard deviation of the control group. We then calculated the predicted normalized fixation bias for each subject based on the coefficients from the multiple linear regression model These predicted values were subtracted from the observed normalized fixation bias to yield a residualized fixation bias measure (that is, variance in fixation bias not accounted for by age, education and value-rating inconsistency).

### Behaviour-based lesion analysis

The Non-Parametric Mapping (NPM, version June 6, 2013) software (freely available at www.mccauslandcenter.sc.edu/mricro/npm/) was used for voxel-based lesion symptom mapping (VLSM) analysis. The residuals of the fixation bias index were used in the VLSM analysis. Voxel-wise comparisons between patients were carried out using nonparametric Brunner–Munzel tests^37^ in all voxels where there were three or more patients with lesion damage. To control for multiple comparisons, a null distribution of Brunner–Munzel *Z*-scores was calculated from the same data set using permutation tests (3,000 permutations)^65^. This method provides an assumption-free means of controlling for multiple comparisons that is also more powerful than commonly used corrections like the Bonferroni method^66^. This test yielded a threshold of *Z*>3.35 (for *P*<0.05) and *Z*>3.48 (for *P*<0.025). Images of the results of this analysis were created using the software MRICron.

### Statistical analysis

Mixed-measures ANOVAs were used to examine the effect of value differences on subjects' fixation properties and reaction times, and to compare groups. Nonparametric Kruskal–Wallis tests were used to test for group differences in value- and brightness-rating consistency, as well as model parameters. *Post hoc* between-subjects *t*-tests, or nonparametric Mann–Whitney *U*-tests, were carried out to test for specific differences between groups where group effects were present. The alpha level of all *post hoc* tests was corrected for multiple comparisons between groups using the Bonferroni method where all pair-wise tests were completed (*α*=0.0083, for *P*=0.05).

Mixed-measures ANOVAs were also used to test for differences in responses to options presented in the contra- and ipsilesional hemifields in patients with unilateral damage. *Post hoc* between-subjects *t*-tests were used to compare between patient groups, with an alpha level corrected using the Bonferroni method (*α*=0.017, for *P*=0.05).

Given that fixation advantage was influenced by the rating difference of options, we corrected for this effect by calculating a measure of fixation advantage that was not predicted by differences in the saliency or rating difference of options. We used multiple linear regression in each subject to calculate a ‘predicted fixation advantage' for each trial based on the rating difference of these options and whether the left option was more salient. We then subtracted the predicted fixation advantage for the left option from the observed fixation advantage in each trial to obtain fixation advantage residuals that were used in all relevant analyses.

GEEs, as implemented in SAS (version 9.4, SAS Institute Inc., Cary, NC, USA) were used to examine subjects' choice behaviour. This analysis is very similar to multiple linear regression, but takes account of the correlation of responses within subjects. Choice of the left option as a binary outcome was modelled as a function of group (a categorical variable referenced to the control group), left–right value-rating difference (an ordinal variable from −2 to 2), fixation advantage to the left versus right option (an ordinal variable with bins for fixation time difference of over 500 ms to the right or left, 150–500 ms more to the right or left, or under 150 ms to either), and saliency difference (a binary variable, greater saliency to the left or right).

Similarly, a GEE was also used to test how fixation bias was influenced by subjects' value ratings. Choice of the option with a greater fixation advantage (binary outcome) was modeled as a function of group (again, referenced to controls), the rating difference of the fixation-advantaged option and the alternative (an ordinal variable from −2 to 2), the magnitude of fixation advantage (an ordinal variable from <150 ms, 150–500 ms or >500 ms) and saliency (a binary variable, greater for fixation-advantaged option or alternative).

In both analyses, we started with a simple GEE model including main effects for each of these variables and then systematically added interactions between each variable and group to test whether lesion damage altered the influence of these variables on choice. The optimal model was selected on the basis of the minimum QIC, which balances fit of the model (based on the maximum-likelihood function) with number of parameters. Supplementary Table 1 provides the QIC statistics for each model and the associated Akaike weights. These weights indicate the relative likelihood of each model based on the QIC statistics. A single odds ratio and 95% CI was computed for each variable, as well as each interaction.

To compare ratings of value and brightness for artworks between the control group and patient groups, we computed the average rating for each artwork in the initial rating task within each group. We then used nonparametric Spearman correlations to test whether the average brightness and value ratings for artworks given by the patient groups followed a similar pattern to the average ratings given by controls.

### Code availability

Computer code used in the analysis of data presented here is available upon request.

## Additional information

**How to cite this article:** Vaidya, A. R. & Fellows, L. K. Testing necessary regional frontal contributions to value assessment and fixation-based updating. *Nat. Commun.* 6:10120 doi: 10.1038/ncomms10120 (2015).

## Supplementary Material

Supplementary Information Supplementary Figures 1-9 and Supplementary Table 1

## Figures and Tables

**Table 1:** Demographic information for controls and prefrontal patients.

**Group**	**Age (years)**	**Sex (M/F)**	**Education (years)**	**BDI-II**	AMNART IQ*	**Lesion Volume (cc)**
CTL (*N*=27)	58.8 (12.9)	9/18	16.4 (3.1)	4.2 (4.9)	121 (5)	—
DMF (*N*=12)	54.1 (10.5)	3/9	14.9 (4.1)	10.4 (6.5)†	117 (7)	15 (3–83)
LF (*N*=8)	59.5 (9.6)	3/5	15.0 (3.5)	6.3 (6.2)	120 (4)	25 (9–96)
VMF (*N*=13)	58.8 (12.0)	5/8	15.8 (2.9)	8.2 (4.9)†	119 (6)	16 (7–77)

Values represent means with standard deviations in parentheses, except for lesion volume where the median and range are provided.

^*^Not all subjects were able to complete the AMNART.

^†^*P*<0.05, two-tailed *t*-test against control scores, uncorrected.

**Table 2:** Performance on neuropsychological screening tests for controls and prefrontal patients.

**Group**	**Posner cueing (uncued-cued) RT left/right (ms)**	**Circle cancellation % missed (left/right)**	**Incidental memory** ***P*** **(correct)**	**Fluency-animals**	**Fluency–F**	**Backwards digit span**	**Sentence comprehension** ***P*** **(correct)**
CTL (*N*=27)	57.8 (45.8)60.5 (38.1)	——	—	—	—	—	—
DMF (*N*=12)	67.5 (57.6)69.7 (51.8)	0.4 (1.0)1.1 (2.3)	0.74 (0.15)*,†	20.0 (8.7)†	11.0 (4.7)†	2.6 (1.0)†	0.98 (0.04)†
LF (*N*=8)	74.6 (33.9)65.6 (47.2)	1.2 (1.9)1.1 (1.8)	0.84 (0.10)	18.9 (7.7)	11.9 (5.9)	2.6 (1.4)	0.93 (0.09)
VMF (*N*=13)	82.4 (37.2)76.0 (40.2)	0.4 (0.9)1.1 (1.3)	0.87 (0.09)	20.0 (3.8)	10.4 (3.9)	3.3 (1.3)	0.98 (0.06)

Values represent means with s.d. in parentheses.

^*^P<0.05, DMF<VMF, two-tailed *t*-test, uncorrected.

^†^Data missing from one patient.

**Table 3:** Multiple linear regression for normalized fixation bias in the control group.

**Predictor**	**Regression coefficient**	**s.e.**	***P***-**value**
Intercept	−0.73	0.86	0.4
Education	−0.52	0.20	0.01
Age	0.04	0.01	0.01
Value-rating inconsistency	−1.55	1.28	0.2

Model adjusted *R*^2^=0.30. *F*-test against constant model: *F*_*3*_,_*2*2_=4.68, *P*=0.01. Education was treated as an ordinal variable (high school or less, undergraduate and graduate level).
