# Supplementary material for: Testing necessary regional frontal contributions to value assessment and fixation-based updating
Source: Nat Commun. 2015 Dec 14;6:10120. doi: 10.1038/ncomms10120 (PMC4682105; doi:10.1038/ncomms10120)
Supplement: Supplementary Information — Supplementary Figures 1-9 and Supplementary Table 1 [file ncomms10120-s1.pdf]

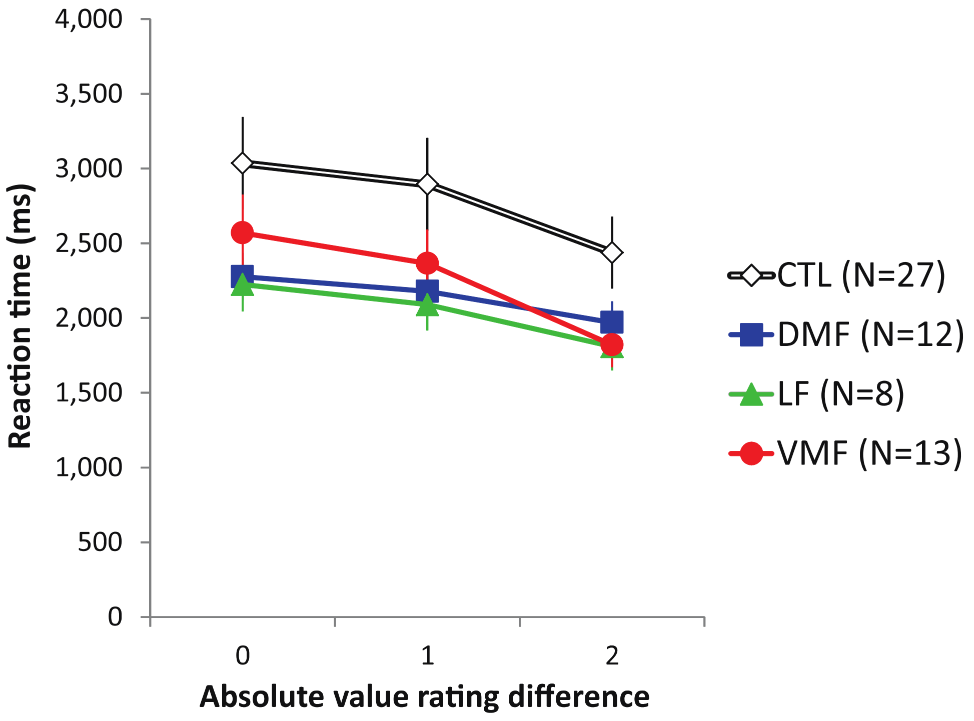

Supplementary Figure 1. Reactions time as a function of the absolute value rating difference of left and right options with outliers (RT greater than 2 standard deviations from subject's mean) removed. Patients and controls responded faster as the absolute value rating difference increased (Mixed measures ANOVA: $F_{2,112}$ = 27.22, $P < 0.0001$). There was no significant main effect of group ($F_{3,56}$ = 1.82, P = 0.1), or interaction between group and value difference ($F_{2,112}$= 1.01 P = 0.4). Error bars represent the SEM.

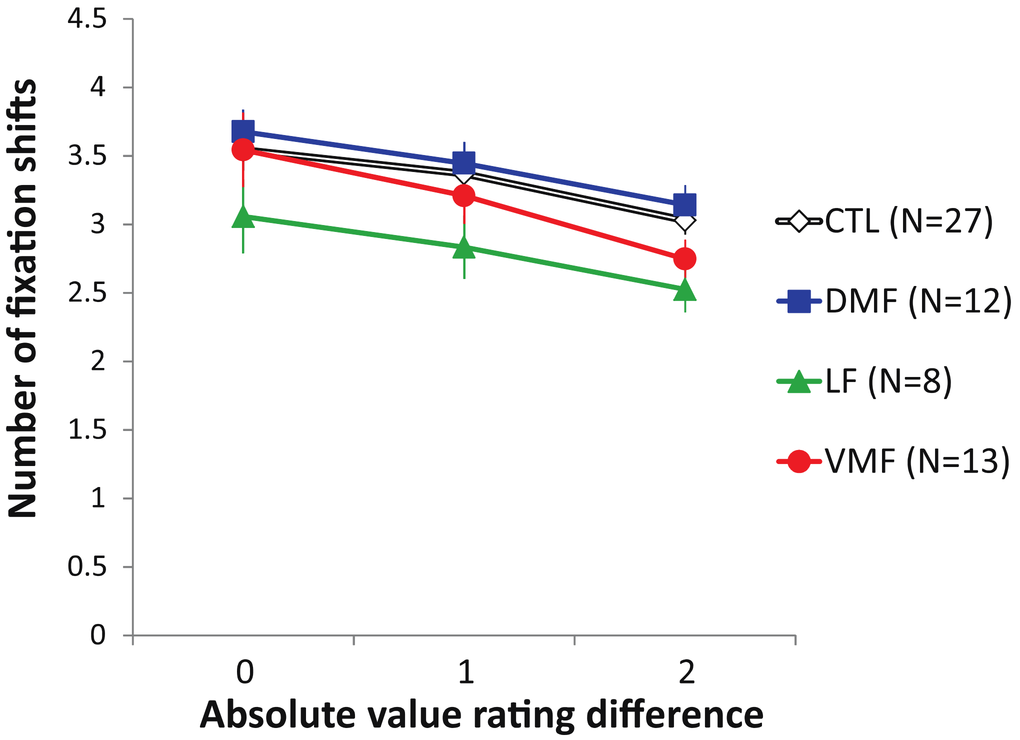

Supplementary Figure 2. Average number of fixations as a function of the absolute value difference between options. There was a significant main effect of absolute value difference, with the number of fixations decreasing as the absolute value difference between options increased (Mixed measures ANOVA: $F_{2,112} = 36.19$, $P < 0.0001$). There was no significant main effect of group on the number of fixation shifts ($F_{3,56} = 1.92$, $P = 0.1$), or interaction between value difference and group ($F_{6,112} = 0.54$, $P = 0.7$).

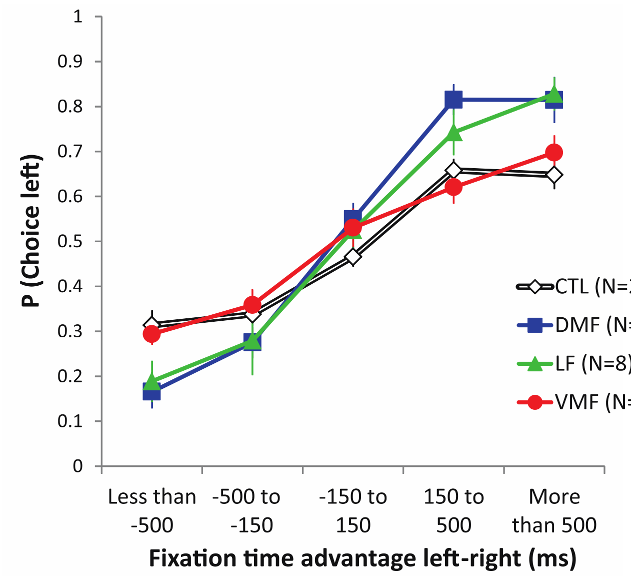

Supplementary Figure 3. Effects of raw fixation advantage on choice of the left option. Using this measure in the GEE, we found that the best model included interactions of group with value rating difference and fixation advantage, but not saliency. There were main effects of value rating difference (OR: 2.65, CI: 2.30-3.05, $P < 0.0001$), fixation advantage (OR: 1.52, CI: 1.39-1.67, $P < 0.0001$) and saliency (OR: 1.22, CI: 1.08-1.38, $P = 0.001$). The DMF group was marginally more likely than controls to choose the left option over the right option overall (OR: 1.29, CI: 1.02-1.65, $P = 0.03$). We found no differences in the effect of value ratings on choices in DMF (OR: 0.86, CI: 0.70-1.06, $P = 0.2$), LF (OR: 1.38, CI: 0.82-2.33, $P = 0.2$) or VMF (OR: 1.22, CI: 0.99-1.52, $P = 0.07$) groups compared to controls. The effect of fixation advantage was greater in the DMF group compared to controls (OR: 1.57, CI: 1.21-2.05, $P = 0.0008$). In contrast, the influence of fixation advantage on choice in the LF group (OR: 1.45, CI: 0.95-2.20, $P = 0.08$) and the VMF group (OR: 1.05, CI: 0.91-1.21, $P = 0.5$) was not significantly different from controls. Error bars represent the SEM.

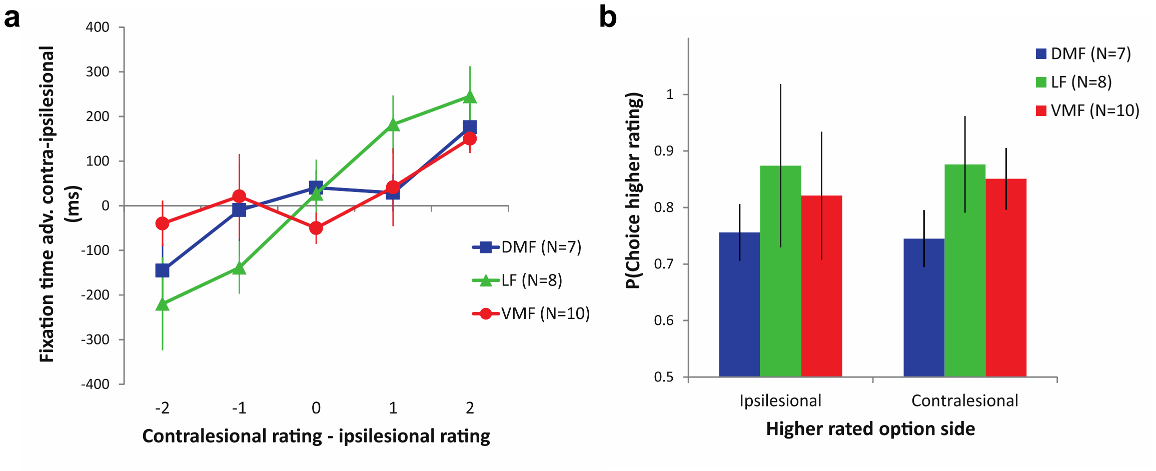

Supplementary Figure 4. Contra- and ipsilesional effects of value ratings on fixation advantage and choices in 25 patients with unilateral damage. (**a**) Fixation time advantage to contralesional-ipsilesional options as a function of the rating difference of these options. There was a significant main effect of rating difference on fixation advantage (Mixed measures ANOVA: $F_{4,88} = 11.23$, $P < 0.0001$). However there was no significant main effect of group ($F_{2,22} = 0.01$, $P = 0.9$), or interaction between group and rating difference ($F_{8,88} = 1.75$, $P = 0.1$). (**b**) Probability of choosing higher rated option on contra- or ipsilesional side. There was a significant main effect of group (Mixed measures ANOVA: $F_{2,22} = 4.00$, $P = 0.03$), with the LF group choosing the higher rated option significantly more often than DMF group (Bonferroni corrected *t*-tests, collapsed across side: $P = 0.009$, two-tailed). Critically, there was no significant effect of side ($F_{1,22} = 0.06$, $P = 0.8$), or interaction between side and group ($F_{2,22} = 0.18$, $P = 0.8$). Error bars represent the SEM.

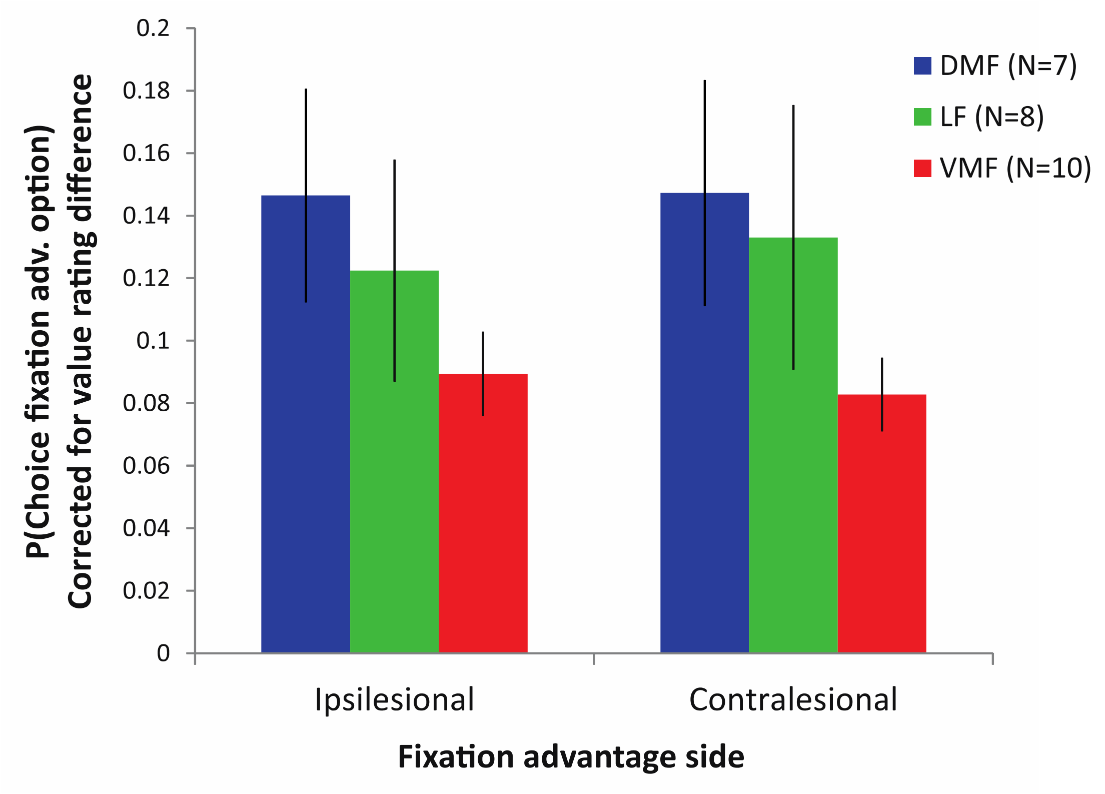

Supplementary Figure 5. Probability of choosing the fixation-advantaged option when it appeared on the contra- or ipsilesional side, corrected for value difference, in 25 patients with unilateral damage. Critically, there was no effect of side ($F_{1,22}$ = 0.12, P = 0.7), or interaction between side and group on the probability of choosing the fixation-advantaged option ($F_{2,22}$ = 1.26, P = 0.3). There was also no main effect of group ($F_{2,22}$ = 1.24, P = 0.3) in this sample of patients. However, the pattern of results was not different from complete patient sample. Error bars represent the SEM.

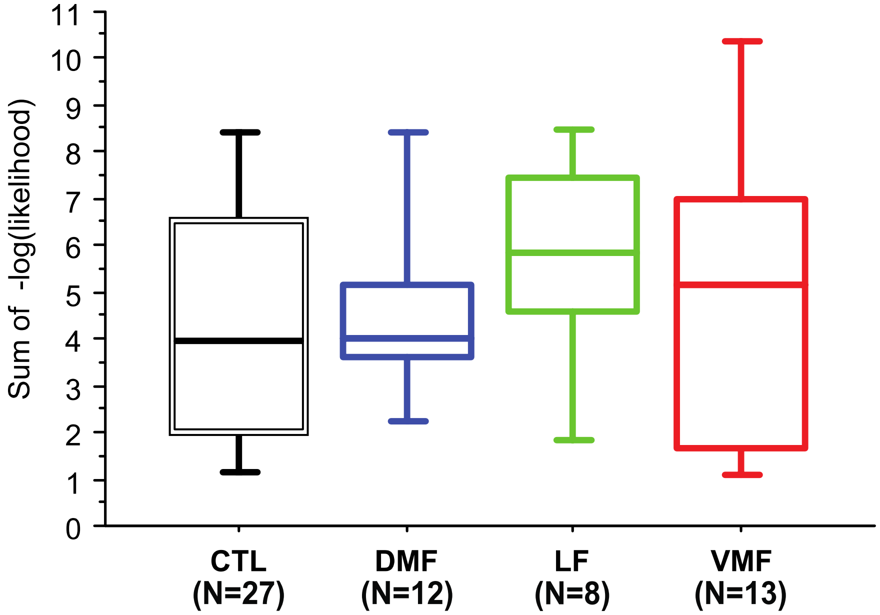

Supplementary Figure 6. Model fitness in each group, calculated as the sum of the negative log likelihood of *P*-values from Kolmogorov-Smirnov tests in the three value difference conditions (0, 1, 2). There was no effect of group status on this measure (Kruskal-Wallis test: $H_3 = 1.53$, $P = 0.7$). Box plots show the 10th, 25th, 50th, 75th and 90th percentiles.

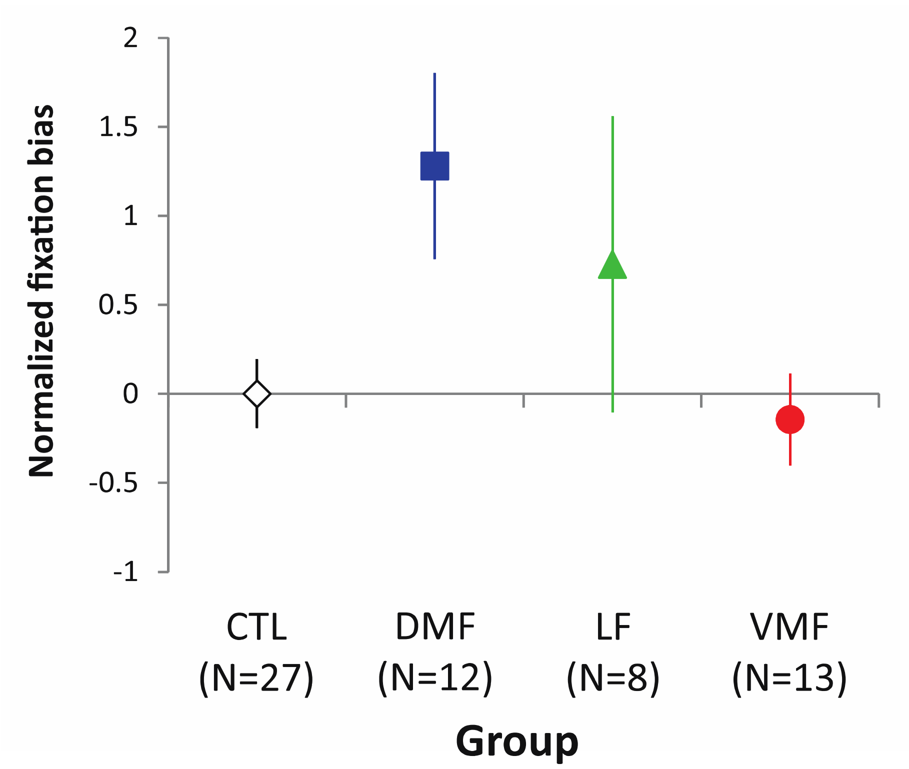

Supplementary Figure 7. Index of fixation bias corrected for value difference and normalized to controls' scores. There was a significant main effect of group on fixation bias (Between-subjects ANOVA: $F_{3,56} = 3.01$, $P = 0.04$). Post-hoc tests found that the fixation time bias of the DMF group approached a significant difference from the control group ($P = 0.07$, corrected, two-tailed) and VMF group (Bonferroni corrected t-test: $P = 0.09$, two-tailed). All other post-hoc comparisons between groups were not close to significant (P's ≥ 0.9 corrected). Error bars represent the SEM.

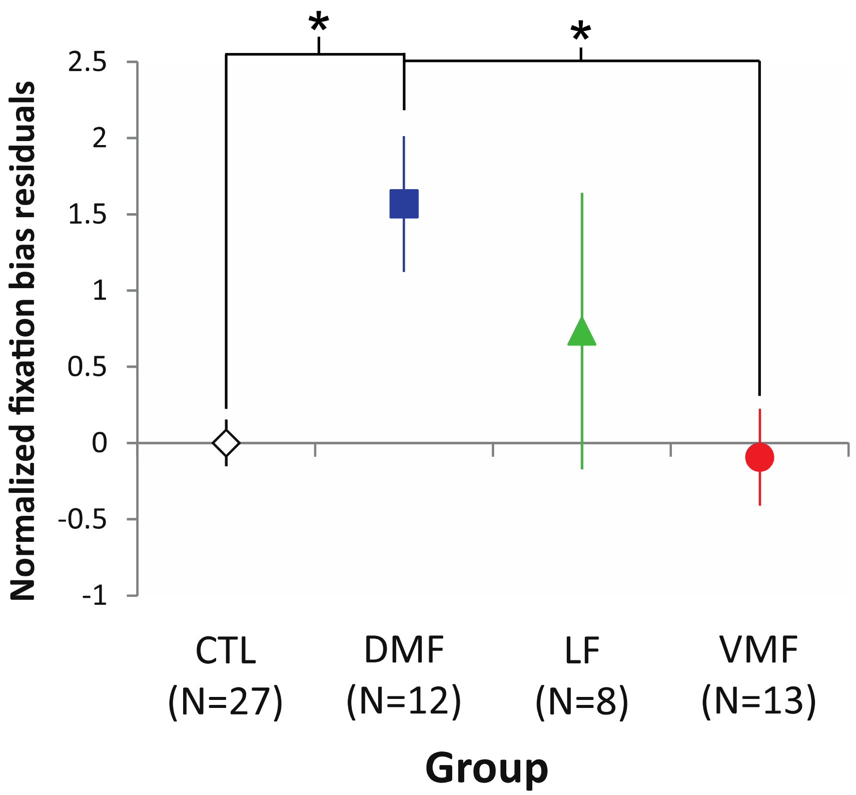

Supplementary Figure 8. Normalized fixation bias residuals after subtracting fixation bias predicted by age, education and value rating difference. There was a significant main effect of group on residualized fixation time bias (Between-subjects ANOVA: $F_{3,56} = 4.45$, $P = 0.007$). Post-hoc tests found that the fixation bias of the DMF group was significantly greater than the control group (Bonferroni corrected *t*-test: $P = 0.01$, two-tailed) and VMF group ($P = 0.02$). There were no other significant post-hoc differences after correction for multiple comparisons (P's ≥ 0.9, corrected). Error bars represent the SEM. * $P < 0.05$, Bonferroni corrected *t*-test, two-tailed.

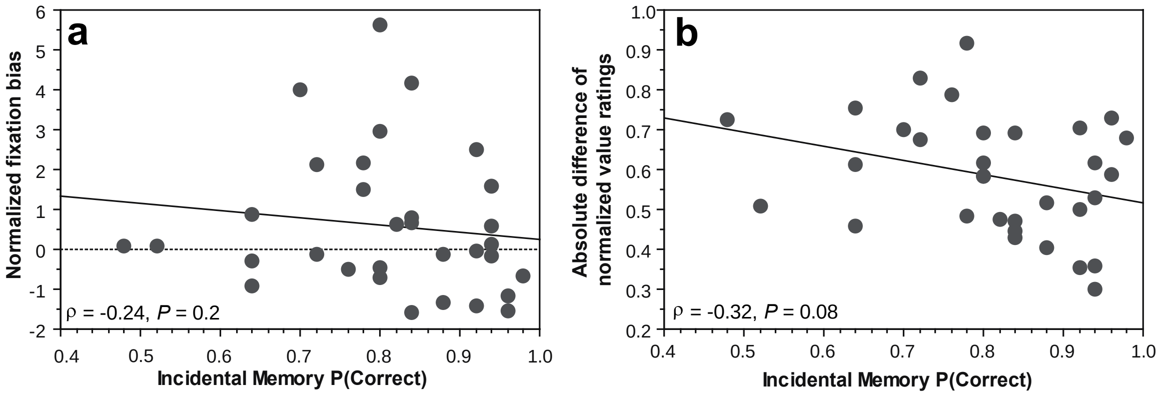

Supplementary Figure 9. Scatterplots showing relationship of incidental memory with (**a**) normalized fixation bias, and (**b**) value rating inconsistency across all patients where incidental memory scores were available (N=32). Spearman correlation coefficient and associated *P* values are shown in the bottom left-hand corner of each panel.

Supplementary Table 1. Quasi-Akaike Information Criteria (QIC) and associated Akaike weights for GEE models.

**Model**	**QIC**	**Akaike Weights**
Choice of left option		
Simple (no interactions)	6139.01	2.17e-8
Group X Rating difference	6132.37	6.02e-7
Group X Fixation advantage	6115.38	2.95e-3
Group X Saliency	6142.55	3.71e-9
Group X Rating difference<br>Group X Fixation advantage	6104.20*	0.79
Group X Rating difference<br>Group X Saliency	6135.23	1.44e-7
Group X Fixation advantage<br>Group X Saliency	6119.20	4.37e-4
Group X Rating difference<br>Group X Fixation advantage<br>Group X Saliency	6106.87	0.21
Choice of fixation-advantaged option		
Simple model	6095.37	0.02
Group X Rating difference	6087.42*	0.98

* Best fit model based on QIC
